# Unleashing anti-tumor immunity: Targeting the autophagy-related protein VPS34 to enhance STING agonist-based therapy

**DOI:** 10.1080/27694127.2024.2370728

**Published:** 2024-07-18

**Authors:** Elisabetta Bartolini, Kris Van Moer, Bassam Janji

**Affiliations:** Tumor Immunotherapy and Microenvironment (TIME) group, Department of Cancer Research, Luxembourg Institute of Health (LIH), Luxembourg City, Luxembourg

**Keywords:** Autophagy, CCL5, CXCL10, CD8 T cells, Immunotherapy, Melanoma, NK cells, PIK3C3, Renal Cell Carcinoma, STING agonist, Type I IFN response, VPS34

## Abstract

The release of CCL5 and CXCL10 is essential for recruiting cytotoxic immune cells into the tumor microenvironment and enhancing the efficacy of cancer immunotherapy. Type I IFNs, particularly IFNβ, activate signaling pathways that induce the expression of these chemokines. In our recent study, we explored the impact and underlying mechanisms of inhibiting the kinase activity of VPS34, a key lipid kinase in the autophagy/endosomal trafficking system, on the expression of CCL5 and CXCL10 in preclinical cancer mouse models. Using NanoString gene expression technology, we analyzed tumors from mice treated with the VPS34 inhibitor SB02024 and demonstrated that the expression of CCL5 and CXCL10 is increased through a cGAS-STING-dependent mechanism within cancer cells. *In vitro*, both pharmacological inhibition and genetic targeting of VPS34 enhance cGAS-STING-mediated expression and secretion of CCL5 and CXCL10 across various tumor cell types. *In vivo*, treatment with the VPS34 inhibitor SB02024 enhances the positive effects of the STING agonist ADU-S100 in melanoma tumor-bearing mice. Thus, our study suggests that VPS34 inhibitors could be used to enhance STING-based anticancer therapies.

**Abbreviations:**

CCL5 (C-C motif chemokine 5); CXCL10 (C-X-C motif chemokine 10); IFN (interferon); VPS34 (vacuolar protein sorting 34); cGAS (cyclic GMP-AMP Synthase); STING (stimulator of interferon genes protein); cGAMP (2′3′-cyclic guanosine monophosphate–adenosine monophosphate).

Autophagy plays a dual role in cancer by acting as either a tumor suppressor or a tumor promoter, depending on the stage of tumor development. During the early stages of tumorigenesis, autophagy prevents the accumulation of damaged organelles and proteins, thereby inhibiting the initiation of cancer. However, in established tumors, autophagy often supports cancer cell survival by providing nutrients and removing damaged cellular components under the hostile conditions.

The interplay between autophagy and tumor immunity has gained significant attention in recent years, and our understanding of autophagy’s role in regulating anti-tumor immunity has driven interest in developing strategies to modulate it for cancer therapy. However, a fundamental question persists: should autophagy be inhibited or activated to improve anti-tumor immunity? There are arguments supporting both the inhibition and activation of autophagy as strategies for enhancing anti-cancer immunity. On the one hand, autophagy stimulates the presentation of tumor antigens by major histocompatibility complex molecules, thereby facilitating the recognition and destruction of tumor cells by cytotoxic T lymphocytes. Moreover, autophagy affects the function of dendritic cells, macrophages, and T cells. For instance, autophagy is required for the processing and presentation of antigens in dendritic cells, while it is essential for the survival and function of T cells. On the other hand, inhibiting autophagy in cancer cells can reprogram the tumor microenvironment by enhancing the secretion of cytokines and chemokines. This drive the recruitment of cytotoxic immune cells to the tumor, transforming “cold” immune-desert tumors into “hot” immune-infiltrated tumors and thereby enhancing the effectiveness of cancer immunotherapy. Furthermore, as a degradative mechanism, autophagy can be exploited by tumor cells to evade immune surveillance by degrading components of the immune system, thereby avoiding destruction by cytotoxic immune cells.

Immunotherapy based on STING agonists has garnered significant interest in cancer treatment due to its ability to trigger a potent innate immune response. The cGAS-STING signaling pathway is activated by agonists or by double- or single-stranded cytoplasmic DNA derived from either tumors or pathogens. This pathway begins with the activation of cGAS, which catalyzes the synthesis of cGAMP from ATP and GTP. Subsequently, cGAMP activates STING at the endoplasmic reticulum and promotes its translocate to the Golgi. At Golgi, STING stimulates *TBK1* (TANK-binding kinase 1) and *IRF3* (interferon regulatory factor 3). Phosphorylated IRF3 dimerizes and translocates to the nucleus, where it induces the transcription *IFN* genes, such as *IFNB1* (**Figure 1**). NF-κB (nuclear factor kappa-light-chain-enhancer of activated B cells) signaling can also be activated by STING.

**Figure 1.**
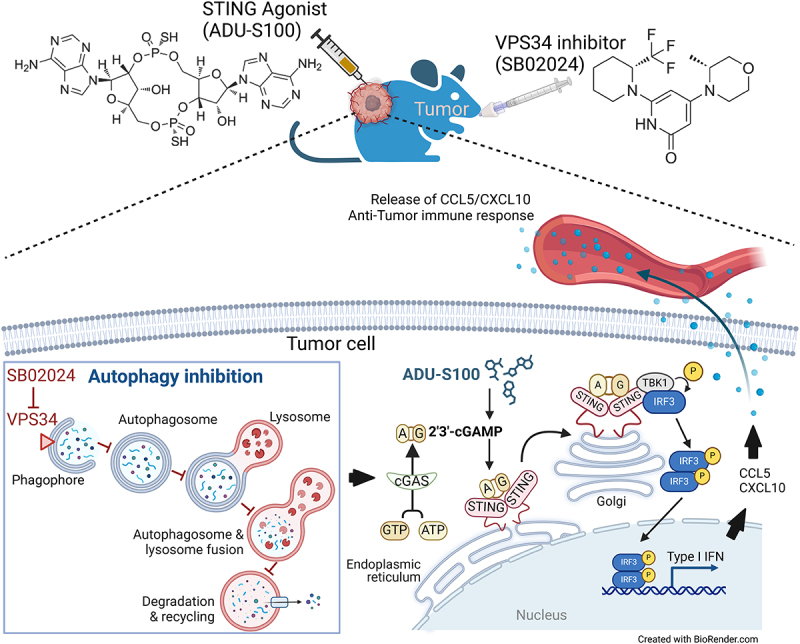
VPS34 inhibition improves cGAS-STING signaling, to trigger the expression and the release of CCL5 and CXCL10, enhancing the anti-tumor immune response. Treatment of tumor-bearing mice with the VPS34 inhibitor SB02024 increases the release of proinflammatory CCL5 and CXCL10 into the bloodstream. This effect also required the concomitant activation of cGAS-STING signaling by the STING agonist ADU-S100. The underlying mechanism is characterized by autophagy inhibition by SB02024, which facilitates cGAS activation by ADU-S100. The synergistic action of SB02024 and ADU-S100 significantly enhances STING pathway activation, leading to a robust Type I IFN response and the secretion of chemokines such as CCL5 and CXCL10. This cascade drives a potent anti-tumor immune response.

Type I IFNs and other pro-inflammatory cytokines are crucial for recruiting activated immune cells like dendritic cells, macrophages, and T cells. By promoting a favorable immune tumor microenvironment, STING agonists have emerged as a promising immunotherapy strategy.

While initial preclinical investigations with STING agonists showed promise, advanced clinical trials have been disappointing results, showing little or marginal improvements in patients response rates. Identifying cellular events that disrupt the STING pathway is crucial for developing combination strategies to enhance clinical outcomes of STING agonists.

We provided preclinical evidence demonstrating the benefits of inhibiting autophagy to enhance the efficacy of STING agonist-based cancer therapy ^1^. Indeed, early findings suggested that autophagy acts as a negative regulator of STING. The underlying mechanism involves the interaction between the autophagy protein BECN1 (Beclin1) and cGAS, leading to the suppression of cGAMP synthesis and the degradation of cytosolic DNA through its interaction with the autophagy cargo protein SQSTM1/p62 (Sequestosome 1). Therefore, autophagy prevents excessive activation of cGAS, thereby avoiding persistent innate immune responses and type I IFN production. Our results support the use of autophagy inhibitors in combination with STING agonists by showing that, in mice bearing renal cell carcinoma, the VPS34 inhibitor SB02024 significantly increased blood levels of the proinflammatory chemokines CCL5 and CXCL10 (**Figure 1**). Tumor transcriptomic analysis from SB02024-treated mice revealed increased expression of gene markers for T cells, Natural killer (NK) cells, macrophages, and neutrophils, which correlated with a notable upregulation of IFN signaling pathways, especially the cGAS-STING pathway. These findings highlight the therapeutic potential of combining VPS34 inhibitors to improve the efficacy of STING agonists. *In vitro* experiments showed that combining VPS34 inhibitors with the STING agonist ADU-S100 significantly enhanced the release of proinflammatory cytokines in various cancer cell types in a cGAS-STING-dependent manner. *In vivo*, the combination of SB02024 with ADU-S100 was tested in B16-F10 melanoma tumor-bearing mice, resulting in a notable reduction in tumor growth and a significant improvement in survival compared to the individual treatments with these drugs.

Our preclinical results were further validated by clinical data from melanoma patients. Compared to individuals with low cGAS expression, those with high cGAS expression demonstrated a significant survival advantage, higher levels of CCL5 and CXCL10, and increased expression of CD8 T cell markers (CD8A and CD8B) and NK cell markers (NCR1 and NCR3). These findings suggest that strategies enhancing the STING pathway could improve immune cell infiltration, likely by inducing proinflammatory cytokines within the tumor microenvironment.

Following clinical disappointments with STING agonists DMXAA and ADU-S100, new candidates including BMS-986301 (NCT03956680) and BI 1387446 (NCT04147234) are currently being evaluated in clinical trials. The outcomes of these studies will be determinant for shaping the future landscape of STING agonists in clinical settings. Our results underline the potential of combining autophagy modulators as a strategy to enhance the effectiveness of next-generation STING agonists. Nevertheless, we believe that combining autophagy modulators in the clinic requires careful and case-by-case evaluation based on specific tumor types and biomarkers to minimize potential adverse effects. While autophagy inhibitors have demonstrated benefits in several situations, other studies have underscored the advantages of autophagy activators for enhancing chemotherapy efficacy, delaying cancer progression in murine models, and reducing regulatory T cell populations. Furthermore, as VPS34 also plays a role in endocytic pathways, it is important to evaluate whether the observed effect on the cGAS-STING pathway relies on inhibiting its autophagic or non-autophagic functions.

In conclusion, deep understanding the complex interaction between cGAS-STING signaling and autophagy is crucial for unlocking their potential in cancer therapy.
